# Penile Pseudoepitheliamatous Hyperkeratosis balanitis: A case report and review in Assiut university urology department 2021

**DOI:** 10.1016/j.eucr.2021.101639

**Published:** 2021-03-17

**Authors:** Abdelrahman Mohamed Abdelkader Osman, Mahmoud Magdy Mahmoud Mohamed Khalil, Mohamed Abdullah Morsy ElGammal, Dalia Ahmed Hamed El-Sers

**Affiliations:** a Department of Urology, Faculty of Medicine, Assiut University, Egypt; b Department of Pathology, Faculty of Medicine, Assiut University, Egypt

**Keywords:** Penis, Conservative, Rare

## Abstract

A 35 years old man present with a skin lesion on his glans complaining of mild irritation. The condition began 3 years ago, he took wrong medication as he was diagnosed as psoriasis, mostly because the disease in it the beginning is very similar and could trick the dermatologist. He was diagnosed by the biopsy taken,Pseudo-epitheliomatous hyperkeratotic and micaceous balanitis. 5-Flouracil was given in combination with oral Acitretin, Dramatic improvement occurred in both the skin lesion and the symptoms associated.

## Introduction

Cutaneous disorder are considered by many patients as a very annoying, distressing and embarrassing condition leading them to seek medical attention. Genital skin disease are very common in almost all age groups. They may be caused by infectious agents or inflammation or allergy or autoimmune or idiopathic. Interfere with sexual functioning, self-image and interpersonal relationships. Some of them are infectious due to sexually transmitted diseases. Many of them are premalignant lesion require immediate intervention. The onset of these disease are variable ranging from acute to chronic. The exact epidemiology is not defined. The treatment methods differ from simple topical medication to radical surgical interventions.

## Case report

**Fig. 1:**
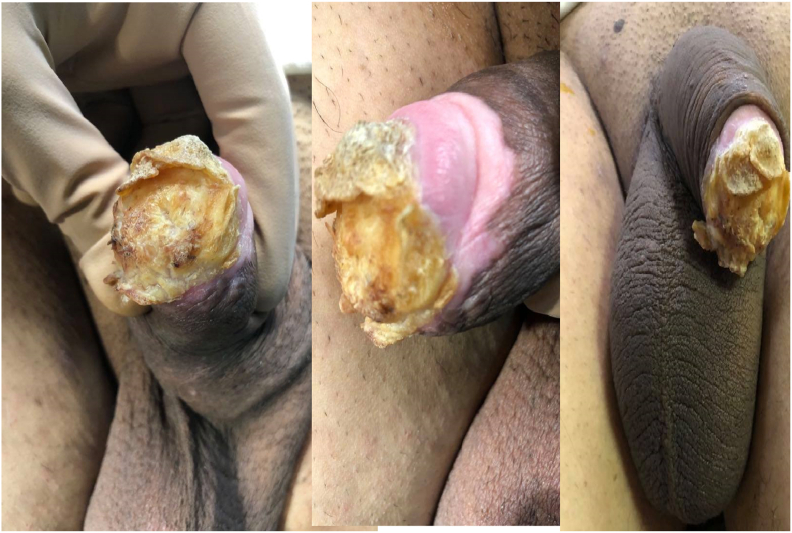
This is the penile lesion before treatment.

A male patient 35 years old married and has 3 offspring, presented to our department complaining of a skin lesion of 3 years duration of insidious onset, he has no family history or significant illness. He stated that there is some pain at micturition also difficulty but of no reduction in the quality of life. Full lab was done, abdominal ultrasound and post-micturition measurement in addition to MRI all normal. By examination no tenderness, a hard yellowish, brownish, scaly nail like lesion no discharge found as shown in Fig. 1.

**Fig. 2:**
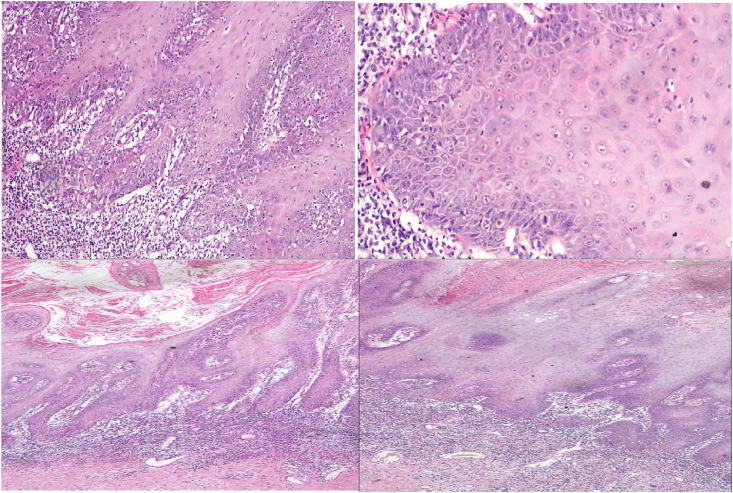
Pathology.

**Fig. 3:**
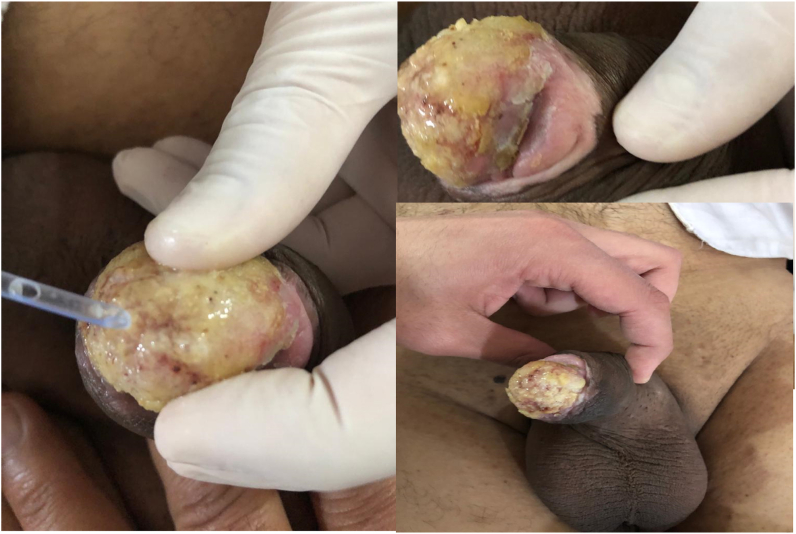
This is the penile lesion after treatment for 2 month, significant improvement is noticed and disappearance of the nail like projection and the glans is gaining its normal appearance.

A wedge shaped biopsy taken from the glans under general anesthesia, we tried to calibrate the urethral meatus with a 12-fr dilator it but passed with resistance so we stopped the maneuver which indicate involvement of the distal urethra. Histopathology revealed hyperplastic stratified squamous epithelium with marked parakeratosis with downward proliferation of squamous cells in sawtooth pattern that don't exceed the lamina propria, the lower third of the epithelium showed mild dysplasia in the form of pleomorphic, large hyper chromatic nuclei with prominent nucleoli and frequent mitoses, band like of lymphocyte mixed with polymorphic nuclear cell infiltrate the lower third of the lesion and subepithelia tissue also dyskeratosis was noticed, in conclusion pseudoepitheliomatous hyperplasia with dysplasia as shown in Fig. 2. Patient was given oral ACITRETIN for one month. Significant improvement in the lesion in addition to the associated symptoms was noticed after the first month. After two month the Urethral stricture is noticed and patient complained of difficulty, patient stated that he discontinued the drugs. He will need a surgical intervention either endoscopically or open to treat this stricture as shown in Fig. 3.

## Discussion

Pseudo-epitheliomatous hyperkeratotic and micaceous balanitis (PKMB) is a rare skin disease, it mainly affect older men who are uncircumcised or recently underwent circumcision, firstly noticed by Lortat-Jacob and Civatte (1961) as a rare, scaling, raised lesion of the glans penis. The disease is slow in its course also it remain localized with no lymph node involvement. The problem with this class of disease is that patients presents late so a conclusive and rapid diagnosis is mandatory.^1^ It is considered by many as a premalignant lesion.^2^ It could transform to either squamous cell carcinoma or verrucous carcinoma.^3^ The exact etiology is not known. Its differential diagnoses could be a penile cutaneous horn and balanitis xerotica obliterans or psoriasis.^4^ Our case is younger in age than the expected age which is 60–70 years. He had an extensive affection of the distal urethra which was mentioned in the case presented by THOMAS FIELDS, but the patient they mentioned had recurrent (PKMB) that developed into a cutaneous horn then transformed later into a malignant lesion after several excisional biopsies.^5^ By many authors conservative treatment is the golden standard in the form of 5-Flourouracil and oral Acitretin other options include surgery and reconstruction also cryosurgery and radiotherapy. The optimal treatment option in penile malignancy is the radical treatment, to avoid local recurrence. The patient desire should be respected so noninvasive methods are used, they carry a huge risk of recurrence and poor oncological outcome, they include topical drugs, laser ablation, Mohs resection, wide local excision and glans resurfacing.

## Conclusion

PKMB is an uncommon and has a special appearance, which is mica-like crusts and keratotic horny mass on the glans penis. Sufficient biopsies are required to obtain subepithelial tissues for the accurate diagnosis of PKMB.

Surgery or topical and oral agents may be used for treatment.
